# Ophiopogonin B sensitizes TRAIL-induced apoptosis through activation of autophagy flux and downregulates cellular FLICE-like inhibitory protein

**DOI:** 10.18632/oncotarget.23647

**Published:** 2017-12-23

**Authors:** Uddin MD. Nazim, Jae-Kyo Jeong, Sang-Youel Park

**Affiliations:** ^1^ Biosafety Research Institute, College of Veterinary Medicine, Chonbuk National University, Iksan, Jeonbuk 54596, South Korea

**Keywords:** ophiopogonin, autophagy, TRAIL, apoptosis, lung cancer cells

## Abstract

Tumor necrosis factor related apoptosis-inducing ligand (TRAIL), a type II transmembrane protein, belongs to the TNF superfamily. Compared to other family members, TRAIL is a promising anti-cancer agent that can selectively induce apoptosis of various types of transformed cells and xenografts, with negligible cytotoxicity against normal tissues. Ophiopogonin B is a bioactive ingredient of Radix *Ophiopogon japonicus*, which is frequently used in traditional Chinese medicine to treat cancer. In this study, we report that Cellular FLICE (FADD-like IL-1β-converting enzyme)-inhibitory protein (c-FLIP) is the key determinant mediating TRAIL resistance in A549 cells and Ophiopogonin B downregulates c-FLIP and enhances TRAIL-induced apoptosis by activating autophagy flux. In addition, treatment with Ophiopogonin B resulted in a slight increase in the conversion of LC3-I to LC3-II and significantly decreased p62 expression levels in a dose-dependent manner. This indicates that Ophiopogonin B induces autophagy flux activation in human lung cancer cells. Inhibiting autophagy flux by applying a specific inhibitor ATG5 siRNA with Ophiopogonin B mediated enhancement of TRAIL effects. These data demonstrate that downregulation of c-FLIP by Ophiopogonin B enhances TRAIL-induced tumor cell death by activating autophagy flux in TRAIL-resistant A549 cells, and also suggests that Ophiopogonin B combined with TRAIL may be a successful therapeutic strategy for TRAIL-resistant lung cancer cells.

## INTRODUCTION

Lung carcinoma is one of the principal reasons of cancer-related death worldwide [1]. After diagnosis, only 17.4% of all lung carcinoma patients live longer than 5 years [2]. In 2012, over 1.8 million people throughout the world had lung cancer, which is estimated to have caused 1.6 million deaths worldwide [3]. Chemotherapy and radiotherapy are the main tools for cancer therapy after surgery. Commencing cancers are infrequently resistant to single method. For this group of patients, combination chemotherapy can extend the 5-year survival rate and prevent recurrence.

Tumor necrosis factor related apoptosis-inducing ligand (TRAIL), also conversant as Apo-2 ligand and TNFSF10, is a type II transmembrane protein that associates to the TNF superfamily [4, 5]. TRAIL is a promising anti-cancer agent that can selectively initiate cell death in various patterns of transformed cells and xenografts, with negligible cytotoxicity against normal tissues [4–6]. TRAIL exerts its pro-apoptotic effect on tumor cells through interaction with membrane receptors, including death receptors. On the contrary, the binding of TRAIL to decoy receptors or osteoprotegerin perpetrates the opposite effect [7]. The interrelation of TRAIL and death receptors recruits Fas-associated protein with death domain and initiator caspase-8, leading to the composition of death-inducing signaling complex [8–10]. In DISC, pro-caspase-8/10 is auto-cleaved to create an active form. This pathway is deactivated by the c-FLIP, which encloses the processing and stimulation of caspase-8 at the level of DISC formation [11]. To date, three c-FLIP isoforms -c-FLIP-long (c-FLIPL), c-FLIP-short (c-FLIPS), and c-FLIPR - have been recognized at the protein level [12–14], and elevated expression of both c-FLIPL and c-FLIPS isoforms were reported in some lung cancers [15].

Ophiopogonin B (OP-B) is a bioactive ingredient of Radix *Ophiopogon japonicas,* which is an evergreen long-lived medicinal herb. It has been used extensively in Southeast Asia to treat pulmonary disease for many years [16]. *Ophiopogon japonicas* contains the following active components: saponins, polysaccharides, and homoisoflavonoids [17]. Ophiopogonin B, which is partitioned from the traditional Chinese medicinal herb Radix *O. japonicus*, has been determined to exert anticancer effects in cervical cancer and human NSCLC [16, 18].

Autophagy is a lysosome-negotiated multi-step degradation scheme that appears in total eukaryotic cells from yeast to mammals [19]. Assembly of ULK1/2-ATG13-FIP200 complex, which causes elevated levels of an isolation membrane named the phagophore, initiates autophagy. The phagophore extends to engulf a variety of intracellular cargo, including Golgi, mitochondria and damaged organelles to form a vesicle named autophagosome. The development of the autophagosome is indefinite on the class III PI3K compound and recruits autophagy-connected genes to enable the elongation and repletion of the autophagosome [20, 21]. The autophagosome solves with an autolysosome where the cargo is degraded and recycled to the cytosol for the defense of basic metabolism [19, 22, 23]. Although the role of autophagy in tumors is complicated and dependent on various aspects such as tumor type, stage, and genetic affection, it definitely plays an important role in cancer biology [24].

Although the anti-cancer benefit of Ophiopogonin B is well known, its synergy with TRAIL and the molecular outcomes involved are recently unclear. Consequently, the destination of this plan was to ascertain benefit of Ophiopogonin B and its synergistic result in combination with TRAIL.

## RESULTS

### Ophiopogonin B sensitizes TRAIL-initiated apoptosis in A549 cells

**Figure 1:**
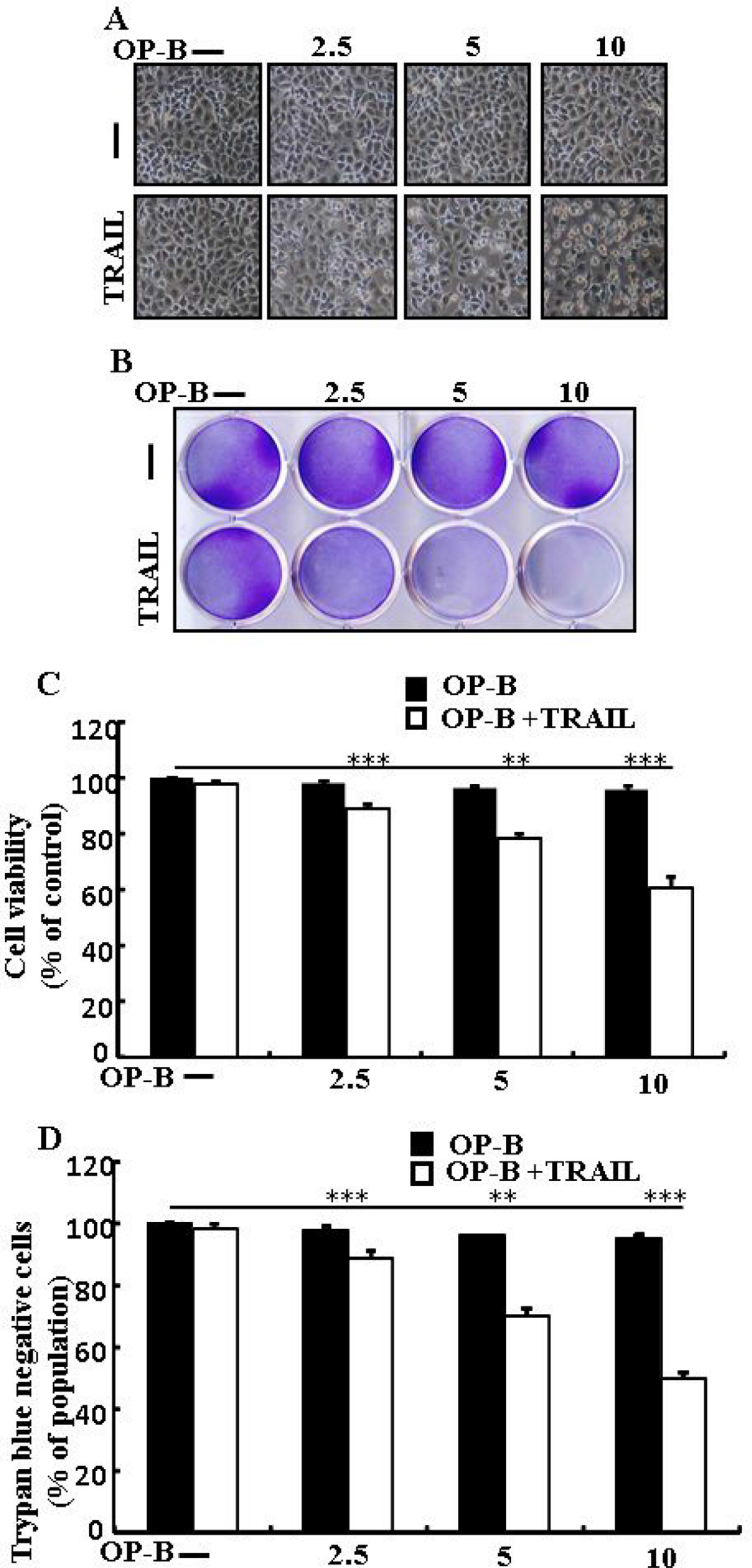
Ophiopogonin B sensitizes TRAIL-initiated apoptosis in A549cells A549cells were pre-incubated with Ophiopogonin B at different doses (0, 2.5, 5, and 10 μM) for 12 h and exposed to TRAIL protein 200 ng/ml for 2 h. (**A**) Cell morphology photographed using light microscope (×100); (**B**) Cell viability was measured with crystal violet assay; (**C**) Bar graph indicating the average density of crystal violet; (**D**) Cell viability was assessed with trypan blue dye exclusion assays. ^**^p < 0.01, ^***^p < 0.001: represent significant differences between control and each treatment group; OP-B: Ophiopogonin B; TRAIL: Tumor necrosis factor (TNF)-related apoptosis-inducing ligand.

We visualized A549 cells using a microscope to supervise the morphological alteration. TRAIL or Ophiopogonin B alone marginally commenced cell death (Figure 1) and no morphological novelty was established. Notwithstanding, combined regimen of TRAIL with different doses of Ophiopogonin B attenuated cell viability compared with Ophiopogonin B or TRAIL (Figure 1A–1D). These data suggest that Ophiopogonin B sensitizes lung adenocarcinoma A549 cells to TRAIL-arbitrated apoptosis.

### Ophiopogonin B induces autophagy and sensitizes cells to apoptosis arbitrated by TRAIL

**Figure 2:**
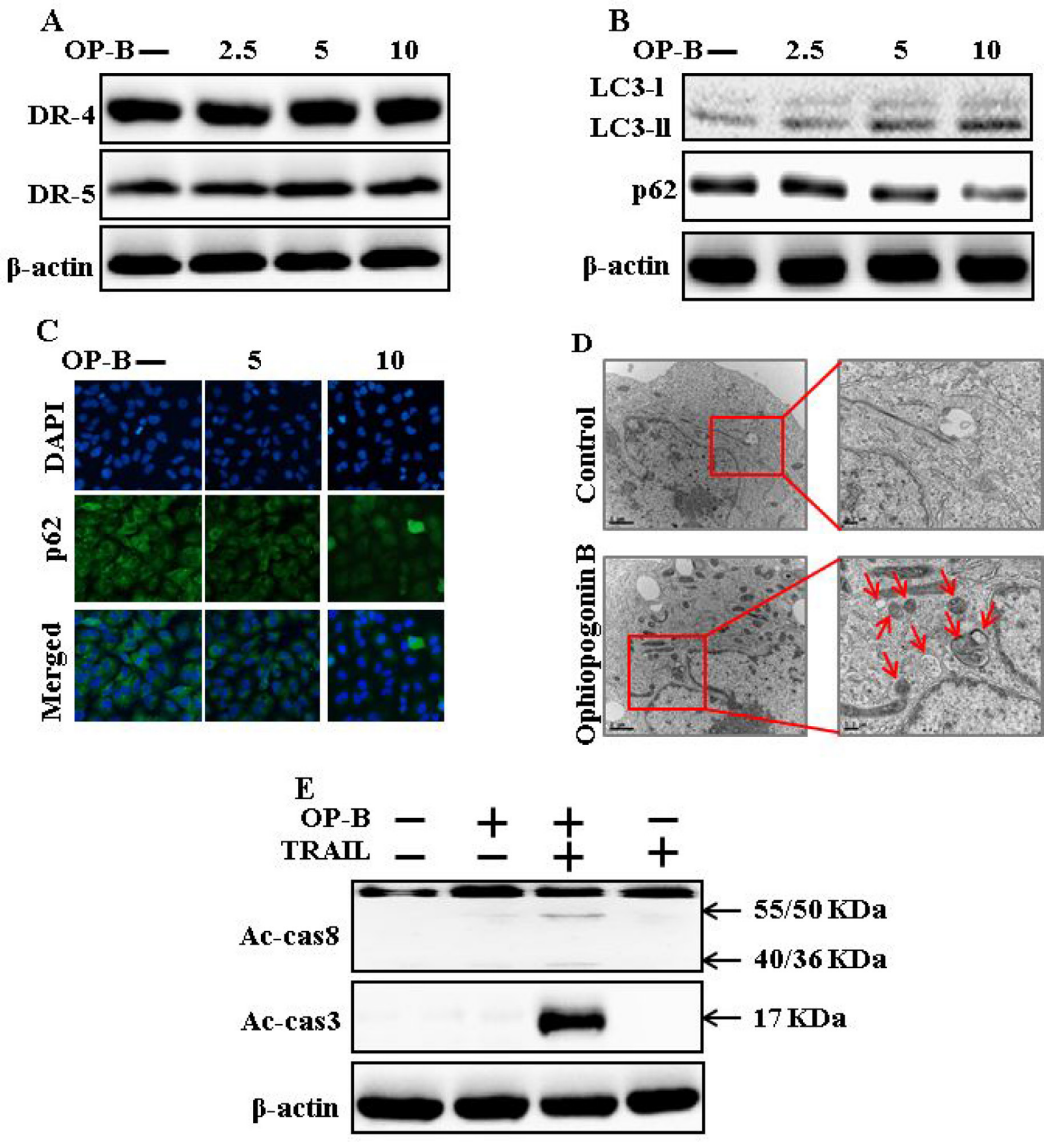
Ophiopogonin B induces autophagy and sensitizes cells to apoptosis arbitrated by TRAIL A549 cells were pre-incubated with Ophiopogonin B at varying doses (0, 2.5, 5, and 10 μM) for 12 h. (**A** and **B**) Western blot for DR-4, DR-5, LC3-II, and p62 proteins was analyzed from A549 cells; (**C**) Cells were immunostained with p62 antibody (green) and assessed in fluorescent view; (**D**) TEM displaysthe ultrastructure of cells treated with Ophiopogonin B (10 μM) for 12 h. Arrows indicate autophagosomes, together with residual digested material and emptyvacuoles; (**E**) Western blot for Ac-cas3 and Ac-cas8 expression levels was conducted with A549 cells. Cells were pre-incubated with Ophiopogonin B (10 μM) for 12 h and exposed toTRAIL protein for an exceeding 1 h. β-actin was used as the loading control. OP-B: Ophiopogonin B; TRAIL: Tumor necrosis factor (TNF)-related apoptosis-inducing ligand; Ac-cas3: Activated caspase 3; Ac-cas8: Activated caspase 8.

As demonstrated in Figure 2A, expression of DR4 and DR5 were unaltered by Ophiopogonin B at any concentration. Nevertheless, LC3-II level was increased and that of p62 attenuated after Ophiopogonin B treatment (Figure 2B). Immunofluorescent staining results also indicated that increasing concentrations of Ophiopogonin B attenuated p62 levels (Figure 2C). A transmission electron microscopy result confirmed that sufficient autophagic and empty vacuoles exposed with Ophiopogonin B treatment (Figure 2D). Combined regimen with Ophiopogonin B and TRAIL sensitized expression of Ac-cas3 and Ac-cas8 (Figure 2E). These findings revealed that Ophiopogonin B could incite autophagy.

### Ophiopogonin B-mediated enhancement of TRAIL-initiated apoptosis is enclosed by attenuation of autophagy

**Figure 3:**
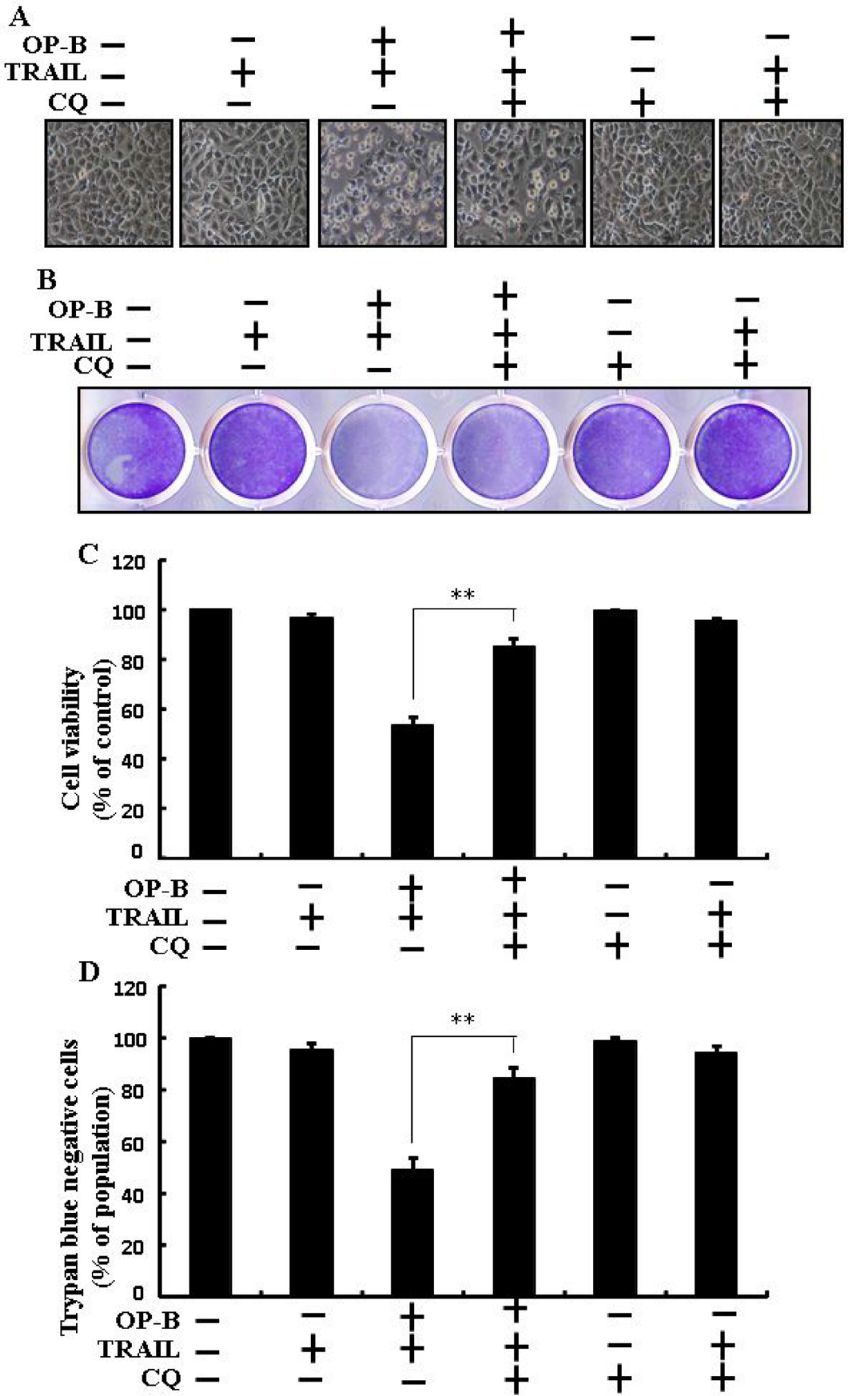
Ophiopogonin B-mediated enhancement of TRAIL-initiated apoptosis is enclosed by attenuation of autophagy Cells were pre-incubated with the indicated Ophiopogonin B (10 μM) doses for 12 h and exposed to TRAIL protein for an additional 2 h. Additional cells were also pre-incubated with autophagy inhibitor chloroquine for 1 h followed by Ophiopogonin B treatment. (**A**) Cell morphology photographed using light microscope (×100); (**B**) Cell viability was measured with crystal violet assay; (**C**) Bar graph indicating average density of crystal violet; (**D**) Cell viability was assessed with trypan blue dye exclusion assays. ^**^p < 0.01:represent significant differences between control and each treatment group; OP-B: Ophiopogonin B; TRAIL: Tumor necrosis factor (TNF)-related apoptosis-inducing ligand; CQ: Chloroquine.

As demonstrated in, co-treatment of chloroquine, Ophiopogonin B and TRAIL enclosed cell death. Morphological consequences confirmed that chloroquine enclosed the cell death outcome (Figure 3A). Co-treatment of chloroquine, TRAIL and Ophiopogonin B significantly sensitized viability with strongly attenuated cell death (Figure 3B–3D). These findings revealed that chloroquine could block Ophiopogonin B-arbitrated apoptosis initiated by TRAIL.

### Attenuation of autophagy encloses TRAIL-arbitrated apoptosis sensitized by Ophiopogonin B via incitation of autophagy flux

**Figure 4:**
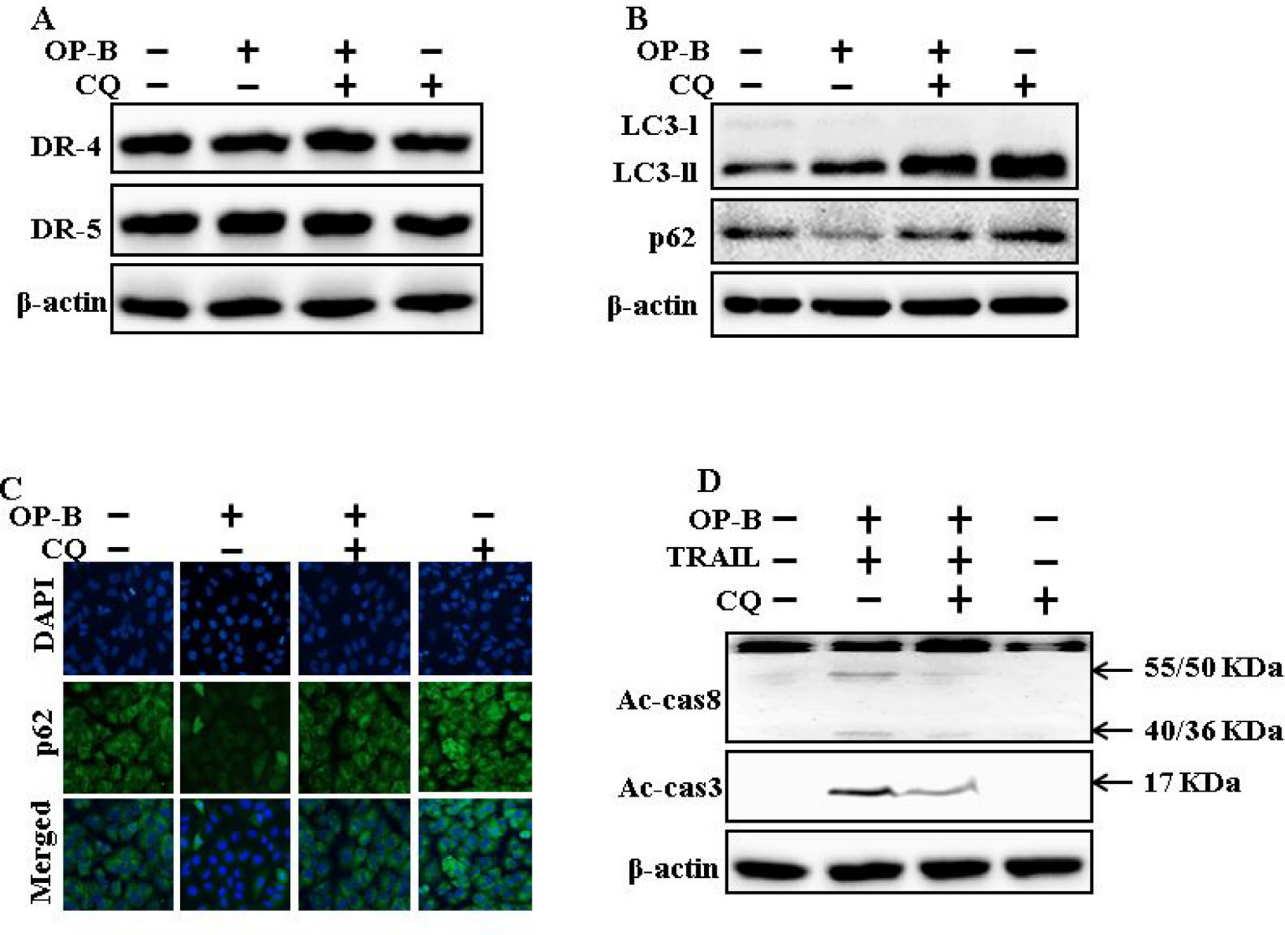
Attenuation of autophagy encloses TRAIL-arbitrated apoptosis sensitized by Ophiopogonin B via incitation of autophagy flux A549 cells were pre-incubated with chloroquine for 1h followed by indicated Ophiopogonin B (10 μM) doses for 12 h. (**A** and **B**) Western blot for DR-4, DR-5, LC3-II, and p62 proteins was analyzed from A549 cells; (**C**) Cells were immunostained with p62 antibody (green) and assessed in fluorescent view; (**D**) Western blot for Ac-cas3 and Ac-cas8 expression levels was conducted with A549 cells. Cells were pre-incubated with the indicated Ophiopogonin B (10 μM) concentrations for 12 h and exposed to TRAIL protein for an additional 1 h. Additional cells were pre-incubated with autophagy inhibitor chloroquine for 1 h, followed by Ophiopogonin B treatment. β-actin was used as the loading control. OP-B: Ophiopogonin B; Tumor necrosis factor (TNF)-related apoptosis-inducing ligand; Ac-cas3: Activated caspase 3; Ac-cas8: Activated caspase 8; CQ: Chloroquine.

The expression of DR4 and DR5 were unaltered by chloroquine or Ophiopogonin B alone, or by combined regimen (Figure 4A). Autophagy adoption was more characterized by the inspection of autophagy flux applying chloroquine. Chloroquine evolved accumulation of LC3-II and a decrease in p62 levels (Figure 4B). Immunofluorescent staining findings also approved that Ophiopogonin B attenuated the p62 protein (Figure 4C). The combined regimen with Ophiopogonin B and TRAIL sensitized the expression of Ac-cas3 and Ac-cas8. Nevertheless, co-treatment of TRAIL, Ophiopogonin B and chloroquine diminished the increase in expression (Figure 4D). These findings revealed that Ophiopogonin B-arbitrated enhancement of TRAIL-initiated apoptosis could be enclosed by chloroquine by autophagy flux incitation.

### Ophiopogonin B-sensitized TRAIL-initiated apoptosis is enclosed by genetic attenuation of autophagy flux

**Figure 5:**
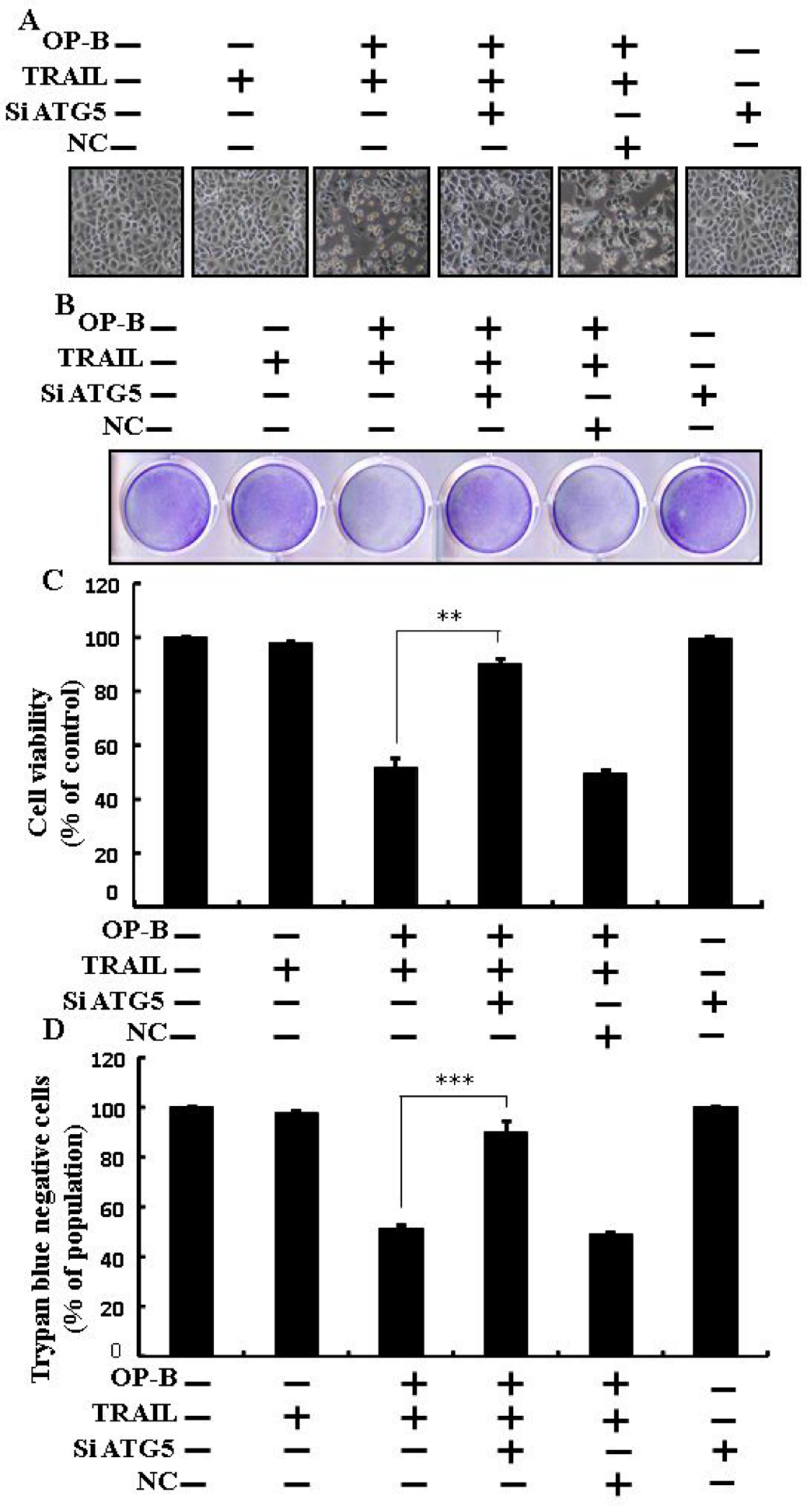
Ophiopogonin B sensitized TRAIL-initiated apoptosis is encloseed by genetic attenuation of autophagy flux A549 cells were pre-incubated with ATG5 siRNA or negative control siRNA for 24 h and then exposed to indicated Ophiopogonin B (10 μM) doses for 12 h with or without TRAIL protein for an additional 2 h. (**A**) Cell morphology photographed using light microscope (×100); (**B**) Cell viability was measured with crystal violet assay; (**C**) Bar graph indicating average density of crystal violet; (**D**) Cell viability was assessed with trypan blue dye exclusion assays.^**^p < 0.01, ^***^p < 0.001:represent significant differences between control and each treatment group. OP-B: Ophiopogonin B; TRAIL: Tumor necrosis factor (TNF)-related apoptosis-inducing ligand; siATG5: ATG5 small interfering RNA; NC: Negative control.

As demonstrated in, co-treatment of ATG5 siRNA, Ophiopogonin B and TRAIL enclosed cell death. Morphological consequences confirmed that ATG5 siRNA enclosed the cell death compared to Ophiopogonin B, TRAIL, and NC (Figure 5A). Co-treatment with TRAIL, Ophiopogonin B, and ATG5 siRNA attenuated cell death with strongly increased viability (Figure 5B–5D). These findings revealed that ATG5 siRNA could block Ophiopogonin B-arbitrated cell death initiated by TRAIL.

### Genetic attenuation of autophagy encloses TRAIL-initiated apoptosis by Ophiopogonin B via autophagy flux incitation

**Figure 6:**
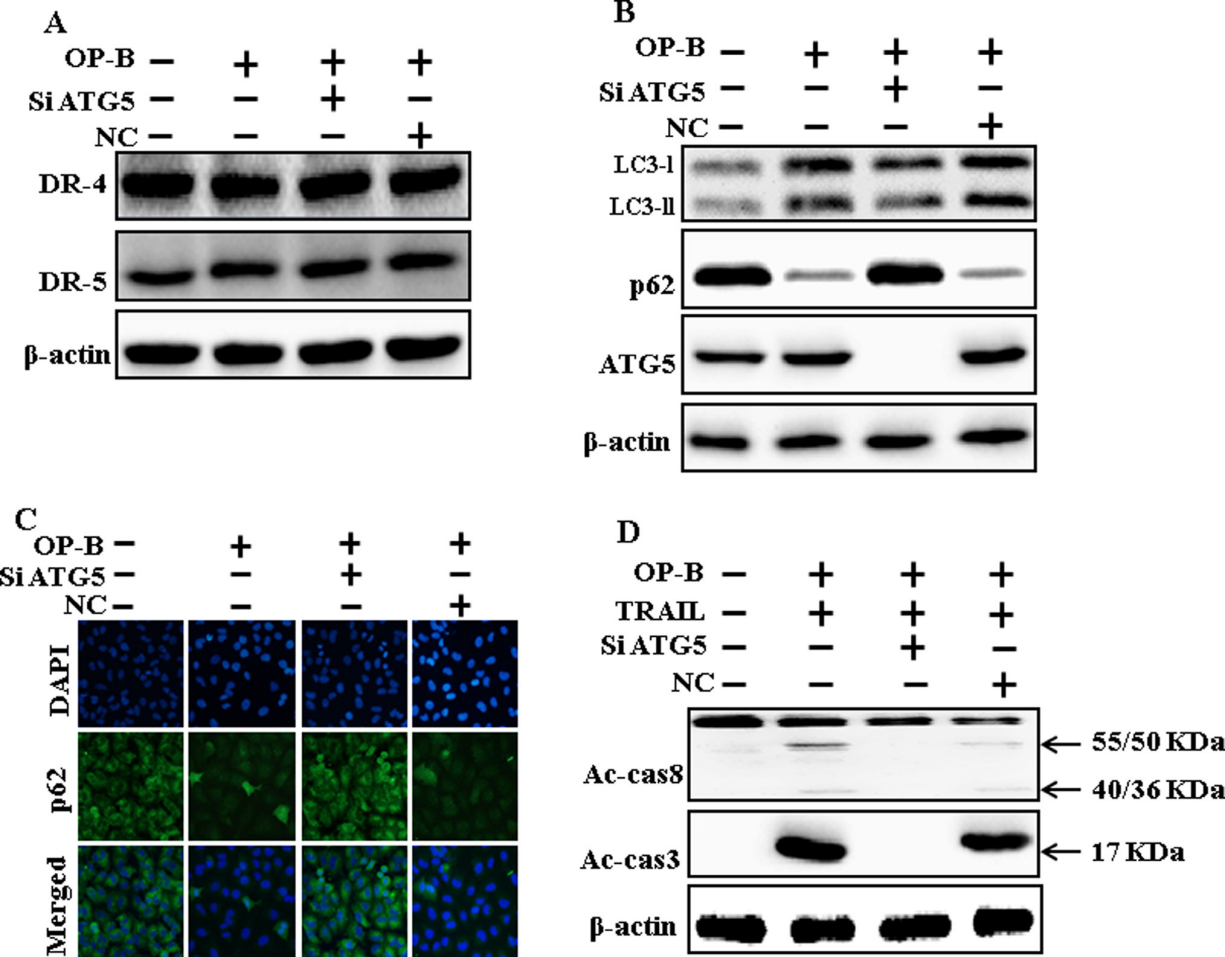
Genetic attenuation of autophagy encloses TRAIL-initiated apoptosis by Ophiopogonin B via autophagy flux incitation A549 cells were pre-incubated with ATG5siRNA or negative control siRNA for 24 h, and then exposed to indicated Ophiopogonin B doses (10 μM) for 12 h. (**A** and **B**) Western blot for DR-4, DR-5, LC3-II, p62 and ATG5 proteins was analyzed from A549 cells; (**C**) Cells were immunostained with p62 antibody (green) and assessed in fluorescent view; (**D**) Western blot for Ac-cas3 and Ac-cas8 expression levels was conducted. A549 cells were pre-incubated with ATG5siRNA or negative control siRNA for 24 h, and then exposed to indicated Ophiopogonin B (10 μM) doses for 12 h with or without TRAIL protein for an additional 1 h. β-actin was used as the loading control. OP-B: Ophiopogonin B; TRAIL: Tumor necrosis factor (TNF)-related apoptosis-inducing ligand; Ac-cas3: Activated caspase 3; Ac-cas8: Activated caspase 8; siATG5: ATG5 small interfering RNA; NC: Negative control.

The expression of DR4 and DR5 were unaltered by Ophiopogonin B alone or by combined regimen (Figure 6A). Knockdown of ATG5 attenuated Ophiopogonin B- initiated LC3-II and markedly increased p62 protein levels (Figure 6B). Immunofluorescent staining findings also revealed this p62 protein level (Figure 6C). Nevertheless, co-treatment with TRAIL, ATG5 siRNA and Ophiopogonin B diminished the increase in Ac-cas3 and Ac-cas8 (Figure 6D). These data confirmed that Ophiopogonin B-arbitrated enhancement of TRAIL-initiated apoptosis could be enclosed by genetic attenuation of autophagy flux.

### Downregulation of c-FLIP by Ophiopogonin B attenuates TRAIL resistance

**Figure 7:**
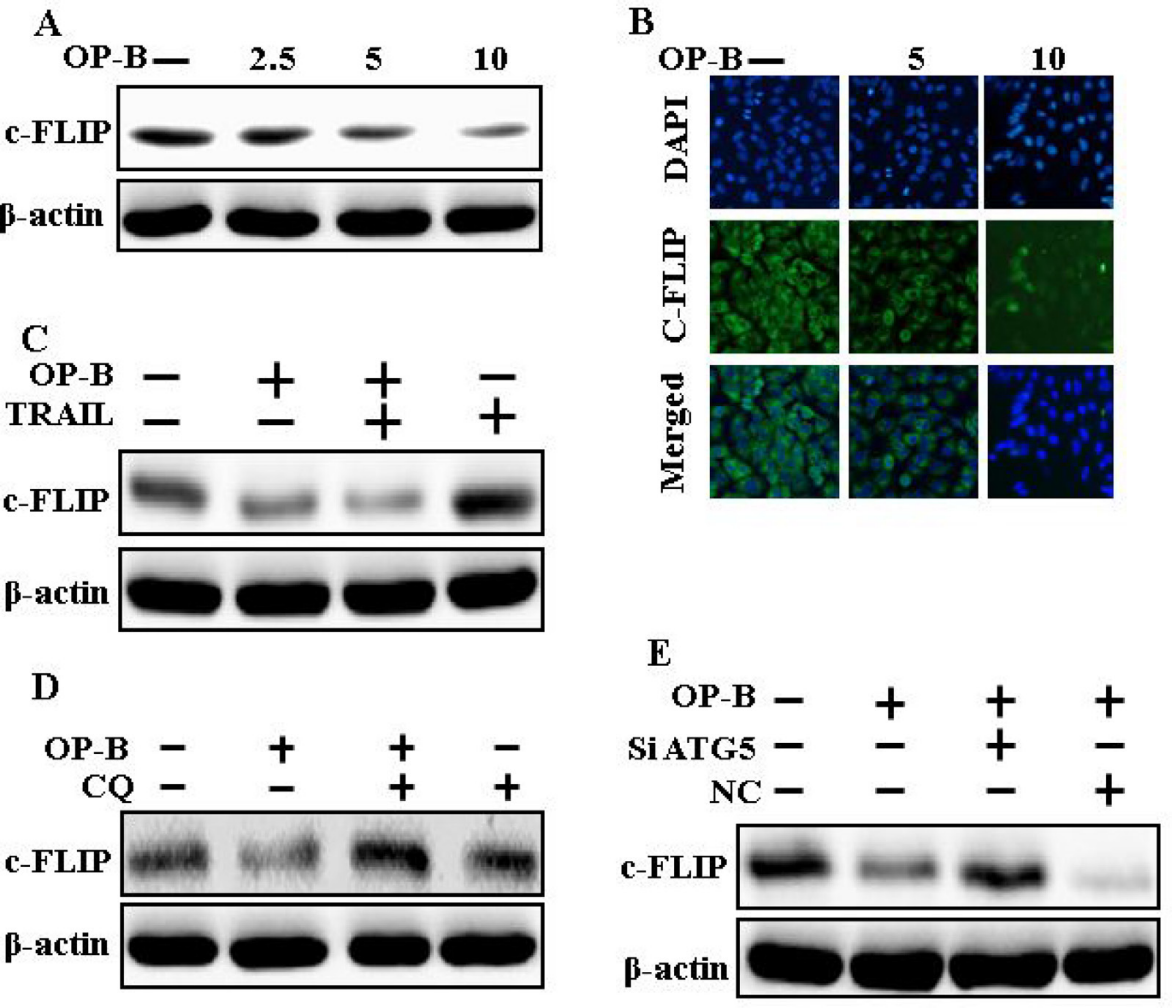
Downregulation of c-FLIP by Ophiopogonin B attenuates TRAIL resistance A549 cells were pre-incubated with varying concentrations of Ophiopogonin B (0, 2.5, 5, and 10 μM) for 12 h and exposed to TRAIL for 1 h. (**A**–**C**) Cells were harvested and assessed by Western blotting and immunofluorescent staining to determine the expression of c-FLIP; (**D**) c-FLIP expression levels emerged on western blot analysis. A549 cells were pre-incubated with chloroquine for 1 h followed by Ophiopogonin B (10 μM) treatment for 12 h; (**E**) Expression levels of c-FLIP emerged on western blot analysis. A549 cells were pre-incubated with ATG5 siRNA or NC siRNA for 24 h followed by Ophiopogonin B (10 μM) treatment for 12 h with or without 200 ng/ml TRAIL for an exceeding1h. β-actin was used as loading control. OP-B: Ophiopogonin B; c-FLIP :cellular FLICE –like inhibitory protein; CQ: Chloroquine; TRAIL: Tumor necrosis factor (TNF)-related apoptosis-inducing ligand; siATG5: ATG5 small interfering RNA; NC: Negative control.

Western blot and Immunofluorescent staining finding revealed that Ophiopogonin B (Figure 7A and 7B) attenuated the expression of c-FLIP. Combined regimen of Ophiopogonin B and TRAIL decreased c-FLIP levels (Figure 7C). Basically, c-FLIP expression was increased by chloroquine when co-treated with Ophiopogonin B (Figure 7D). Furthermore, combined regimen of ATG5siRNA and Ophiopogonin B increased c-FLIP levels (Figure 7E). These finding revealed that Ophiopogonin B downregulates c-FLIP and sensitizes TRAIL-initiated apoptosis by inciting autophagy flux.

### Ophiopogonin B sensitizes TRAIL-initiated apoptosis in HCC-15 and Calu-3 cells

**Figure 8:**
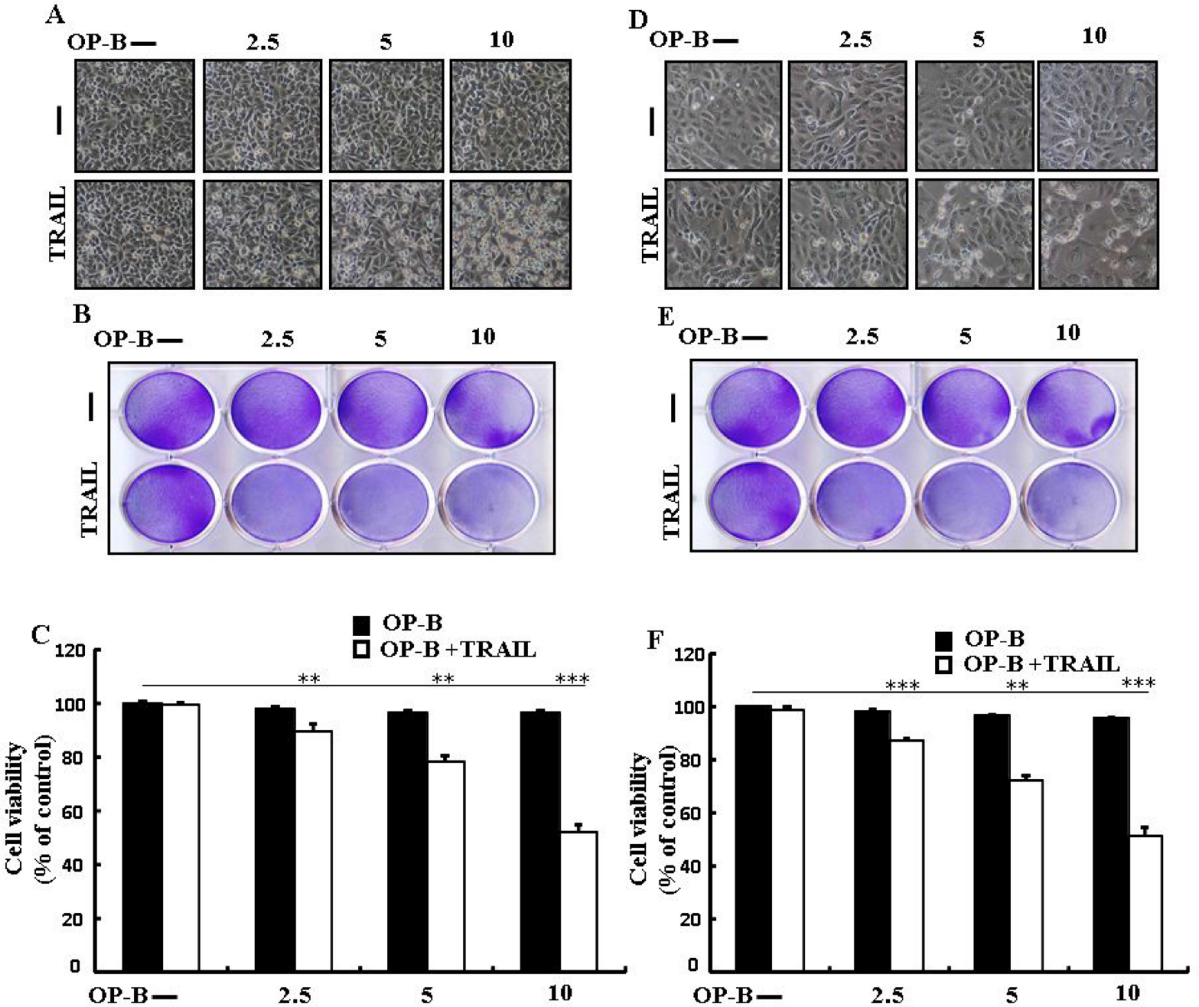
Ophiopogonin B sensitizes TRAIL-initiated apoptosis in HCC-15 and Calu-3 cells HCC-15 and Calu-3 pre-incubated with varying concentrations of Ophiopogonin B (0, 2.5, 5, and 10 μM) for 12 h and exposed to TRAIL for 2 h. (**A** and **D**) Cell morphology photographed under light microscope (×100); (**B** and **E**) Cell viability determined with crystal violet assay; (**C** and **F**) Bar graph showing the average density of crystal violet dye; ^**^p < 0.01, ^***^p < 0.001: represent significant differences between control and each treatment group; OP-B: Ophiopogonin B; TRAIL: Tumor necrosis factor (TNF)-related apoptosis-inducing ligand.

We visualized these cells using a microscope to supervise the morphological alteration. Treatment of TRAIL or Ophiopogonin B alone marginally commenced cell death (Figure 8). Moreover, combined regimen of TRAIL with Ophiopogonin B strongly attenuated viability compared with TRAIL or Ophiopogonin B alone (Figure 8B, 8C, 8E, and 8F). These finding revealed that Ophiopogonin B significantly elevated TRAIL-initiated apoptosis in HCC-15 and Calu-3 cells.

## DISCUSSION

Drug resistance is a major barrier in anticancer chemotherapy. Combined chemotherapy using drugs with disparate mechanisms of function may raise anticancer efficacy [25]. Ideal agents for chemotherapy are those that can discriminately kill cancer cells without harming normal cells. TRAIL is a dynamic chemotherapeutic candidate because of its tremendous anticancer mobility in diverse cancer characters and it asserts minimum cytotoxic outcomes on the vast majority of normal cells [26, 27]. TRAIL has a significant advantage in its selectivity for targeting tumor cells owing to their comparatively higher expression of death receptors than normal cells [6]. Cancer cells acquire resistance by downregulating death receptors and upregulating anti-apoptotic proteins including c-FLIP [28]. Ophiopogonin B, a natural product derived from *Ophiopogon japonicus*, has been widely used in Chinese traditional medicine [29]. Autophagy is cellular degradation process by which cytoplasmic components and organelles are enveloped into the autophagosome and transported into the lysosome to assimilate cytoplasmic debris and maintain cellular homeostasis [30]. Formation of the autophagosome and LC3-I-phospholipid links LC3-II. P62 is accumulates in autophagosomes by specific interaction with LC3, and is easily decayed during autophagy. Suppressing autophagy findings in quick aggregation of p62, and attenuated p62 linked by inciting autophagy [31]. Autophagy maintains a dual function in apoptosis, acting as either a suppressor or a promoter of apoptosis [32].

Recently shown that A549 cells were particularly resistant to TRAIL-initiated apoptosis [33]. In our research focused that TRAIL or Ophiopogonin B alone induced apoptosis slightly or not at all (Figure 1). Some papers have revealed that Ophiopogonin B attenuated cancer cell propagation and adoption of autophagy [16]. Our findings indicated that LC3-II level was elevated and that of p62 was attenuated after Ophiopogonin B regimen (Figure 2). Our findings also confirmed that the diacritic pharmacological (Figures 3 and 4) and genetic autophagy blocker enclosed Ophiopogonin B-arbitrated apoptosis initiated by TRAIL (Figures 5 and 6). Some reports have demonstrated that drug-induced autophagy and downregulation of c-FLIP promotes apoptosis in lung adenocarcinoma cells [34, 35]. Our results also suggested that Ophiopogonin B downregulates c-FLIP and could sensitize TRAIL-initiated cell death by inciting autophagy flux (Figure 7). In summary, attenuation of c-FLIP by Ophiopogonin B sensitizes TRAIL-initiated apoptosis in A549 tumor cells via autophagy flux. Combined regimen with Ophiopogonin B and TRAIL could be a moderate therapeutic method for careful treatment of some TRAIL-resistant cancers.

## MATERIALS AND METHODS

### Cell culture

Cancer cells originating from human lung (A549,HCC-15andCalu-3)tumors were attained from the American Type Culture Collection (Global Bioresource Center, Manassas, VA, USA). Cells were maintained in RPMI-1640(Gibco BRL, Grand Island, NY, USA) medium bearing 10% fetal bovine serum and 100 μg/ml penicillin-streptomycin. Cells were cultured at 37°C and 5% CO_2_ in a humidified incubator.

### Reagents

Recombinant Ophiopogonin B was purchased from chem faces (CheCheng Rd, WETDZ, Wuhan, Hubei 430056, PRC), chloroquine (20 μM) was purchased from Sigma-Aldrich (St. Louis, MO, USA). Recombinant TRAIL (200 ng/ml) was acquired from Abfrontier (Geumcheon-gu, Seoul, South Korea).

### Cell viability assay

A549, HCC-15and Calu-3 cells were plated at 1.0 × 10^4^ cells/well in 12-well plates and incubated at 37°C for 24 h. The A549cells were pretreated with Ophiopogonin B in a dose-dependent manner (0, 2.5, 5, and 10 μM). After pretreated with different doses of Ophiopogonin B for 12 h and were treated with TRAIL protein for an additional 2 h. Additional cells were also pretreated with chloroquine (20 μM) for 1 h, followed by Ophiopogonin B treatment. Cell morphology was examined by photographs under the inverted microscopy (Nikon, Japan). Cell viability was determined applying crystal violet staining method as previously described [25].

### Trypan blue exclusion assay

The number of cell viability was investigated by trypan blue dye exclusion assay (Sigma-Aldrich) using a hemocytometer.

### Western blot assay

A549 cell lysates were prepared by harvesting, washing in cold PBS, resuspending in lysis buffer followed by sonication. Proteins (35 μg) were resolved by 10%–15% SDS gels and transferred to a nitrocellulose membrane, and analyzed by western blotting as described previously [26]. The antibodies were used : LC3 (Novus Biologicals, Littleton, CO, USA), DR-4, DR-5, and ß-actin Sigma-Aldrich (St. Louis, MO, USA), p62ATG5, cleaved caspase-3(Cell Signaling Technology, Danvers, MA, USA), c-FLIP (Enzo life sciences, USA), cleaved caspase-8 (BD pharmingen, USA).

### Immunofluorescent staining

A549 cell lines cultured on glass coverslips positioned on a 24-well plate. The cells were washed with PBS and adjusted with 4% paraformaldehyde for 15 min at room temperature. Following this, Cells were then washed twice with ice-cold PBS, blocked with 5% FBS in Tris-buffered saline with Tween, and incubated with monoclonal antibodies against p62,c-FLIP at room temperature for 24 h. Unbound antibody was removed with PBS wash ( three times) and Cells were then incubated again with secondary antibody at room temperature for 2 h in the dark. Finally, cells were mounted with DakoCytomation fluorescent mounting medium and visualized via a fluorescence microscopy.

### TEM (Transmission Electron Microscopy) analysis

TEM samples were examined by Transmission Electron Microscope (JEM-2010, JEOL) installed in the Center for University-Wide Research Facilities (CURF) at Chonbuk National University. After fixation ofA549 cell samples in 2% glutaraldehyde and 2% paraformaldehyde in 0.05 sodium cacodylate buffer, specimens were post fixed in 1% osmium tetroxide, dehydrated in graded ethanol and propylene oxide. A549 cells were embedded in Epoxy resin. Ultrathin sections were cut on an LKB-III ultratome and were stained with 0.5% uranyl acetate and lead citrate. The images were taken on a Hitachi H7650 electron microscope at an accelerating voltage of 100 kV.

### RNA interference

A549 cells were transfected with ATG5-specific small interfering RNA (siRNA; oligo ID HSS114103; Invitrogen, Carlsbad, CA, USA) using Lipofectamine2000 according to the manufacturer’s instructions. After 36-h post transfection, the knockdown efficiency at protein level was observed by immunoblotting and cell viability test. Nonspecific siRNA was used as a negative control.

### Statistical analysis

All data are expressed as means ± standard deviation (SD) and were compared using the Student’s *t*-test, analysis of variance and the ANOVA Duncan test using SAS statistical package (SAS Institute, Cary, NC, USA). Statistical significance was indicated by a *P* value less than 0.01 (^**^), or 0.001 (^***^).
